# Correction to: Preservation of dendritic spine morphology and postsynaptic signaling markers after treatment with solid lipid curcumin particles in the 5xFAD mouse model of Alzheimer’s amyloidosis

**DOI:** 10.1186/s13195-022-00980-2

**Published:** 2022-03-10

**Authors:** Panchanan Maiti, Zackary Bowers, Ali Bourcier-Schultz, Jarod Morse, Gary L. Dunbar

**Affiliations:** 1 Field Neurosciences Institute Laboratory for Restorative Neurology, Central Michigan University, Mt. Pleasant, MI 48859 USA; 2 Program in Neuroscience, Central Michigan University, Mt. Pleasant, MI 48859 USA; 3 Department of Psychology, Central Michigan University, Mt. Pleasant, MI 48859 USA; 4 Field Neurosciences Institute, Ascension St. Mary’s Hospital, Saginaw, MI 48604 USA; 5 College of Health and Human Services, Saginaw Valley State University, Saginaw, MI 48710 USA

**Correction to: Alz Res Therapy 13, 37 (2021)**

**https://doi.org/ 10.1186/s13195-021-00769-9**

Following the publication of the original article [1] the authors noticed errors in the published Figs. 2, 4 and 5. The original article [1] has been updated.

**Fig. 2:**
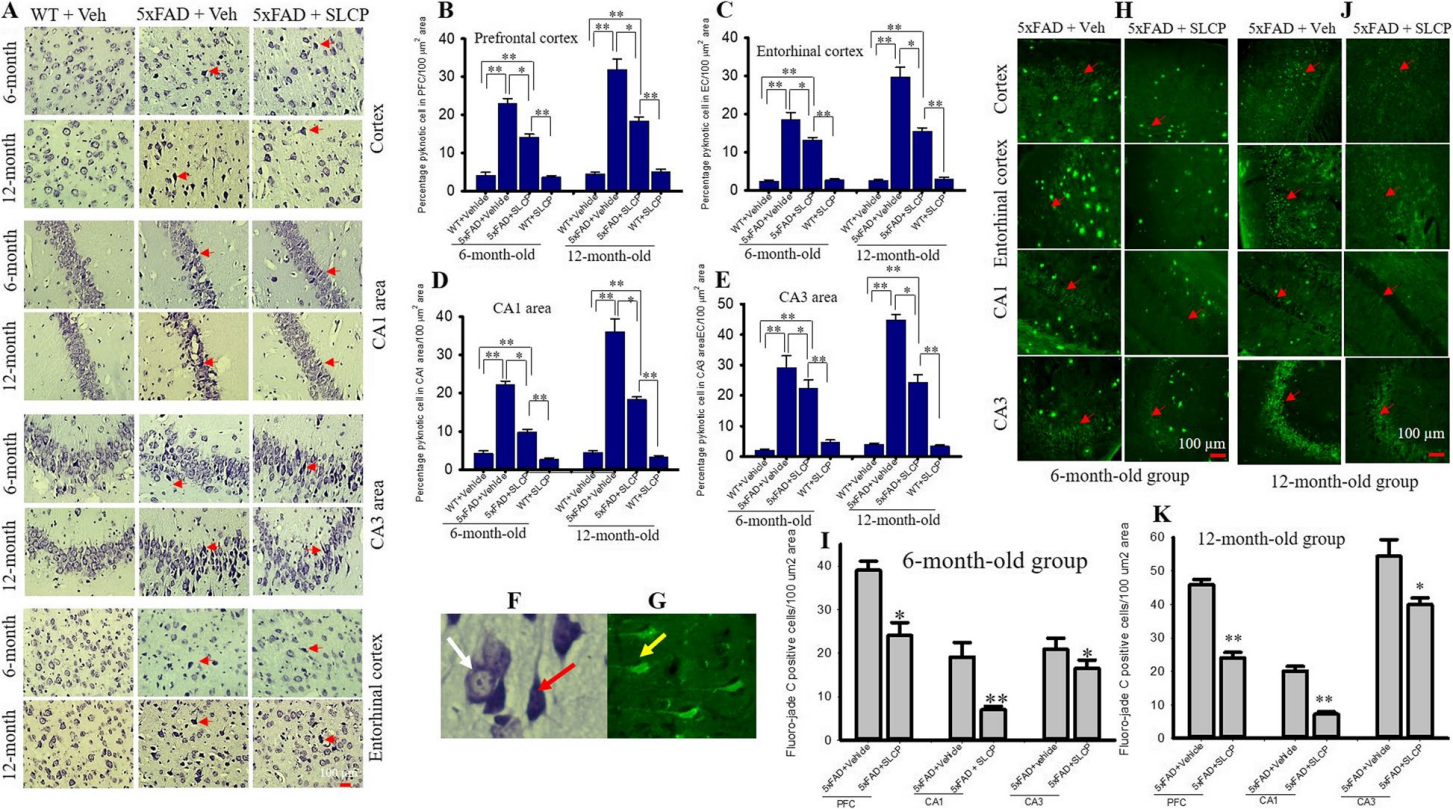
SLCP treatment decreased pyknotic cells and degenerated neurons in PFC and hippocampus and entorhinal cortex of 5xFAD mice. Six and twelve-month-old 5xFAD and age-matched control mice were treated with SLCP (100 mg/kg) or vehicle for 2 months at which they were euthanized, and their brains were perfused with 4% paraformaldehyde. The brains were embedded in paraffin and cut on a rotary microtome into 5-μm coronal sections which were stained with 0.1% cresyl violet. Images were taken through compound light microscope using 40x objectives (total magnification 400x). There was a significant increase in the percentage of pyknotic cells in the PFC (**a**, **b**), and in the EC (**a**, **c**) and CA1 (**a**, **d**) and CA3 (**a**, **e**) areas of hippocampus of the vehicle-treated 5xFAD mice, but these increases were mitigated by SLCP treatments. **f** Image with the white arrow indicating normal and the red arrow indicating pyknotic neurons. **h**–**k** Forty-micron coronal sections were stained with fluoro-jade C (FJC) solution (0.0001%). Images were taken using a fluorescent microscope with a 20x objective (total magnification = × 200). There were significant increases in the number of FJCs in PFC, and in the CA1 and CA3 areas of the hippocampus in the vehicle-treated 5xFAD mice in both 6- (**h**, **i**) and 12-month-old (**j**, **k**) mice, whereas SLCP treatment prevented these increases. g Yellow arrow indicating FJB positive degenerated neuron. *p < 0.05, **p < 0.01 in comparison to WT + vehicle, 5xFAD + SLCP, and WT + SLCP. Red arrows indicate FJC-positive degenerated neurons. Large fluorescent signals are Aβ plaques. Scale bar = 100 μm and is applicable to all images

**Fig. 4:**
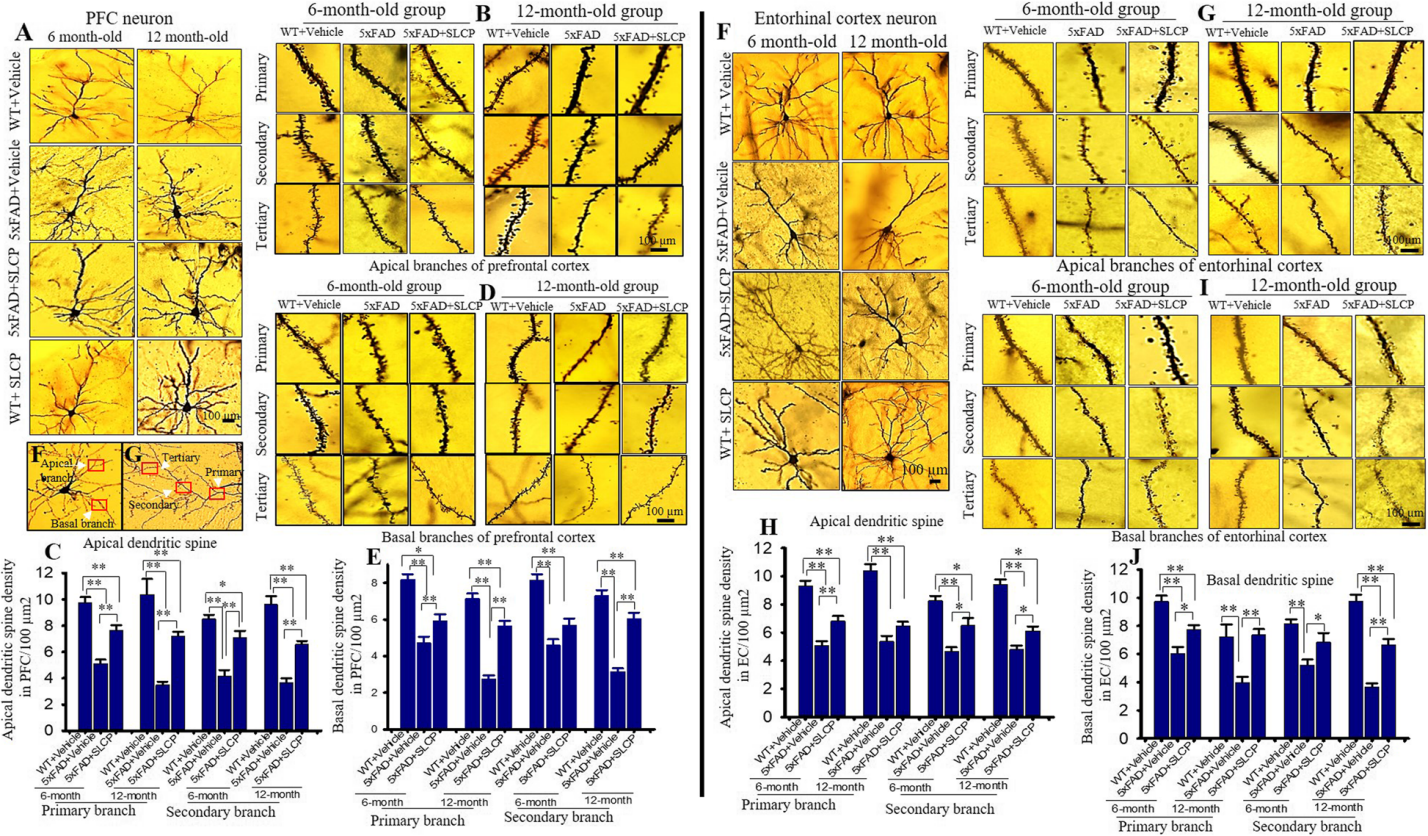
SLCP treatment prevented abnormal dendritic arborization and loss of dendritic spines in the PFC and entorhinal cortex of 5xFAD mice. Six- and twelve-month-old 5xFAD and age-matched control mice were treated with SLCP (100 mg/kg) or vehicle for 2 months and then their brains were extracted and stained with Golgi-Cox stain over a 2-week period. Coronal sections (120 μm) were stained with 75% ammonium solution and 1% sodium thiosulphate. Cortical pyramidal neurons (layer II-III), along with dendritic spines from apical and basal branches (primary, secondary, and tertiary) were imaged using × 40 and × 100 objectives, respectively. **a** Representative images from layer II cortical pyramidal neurons processed with Golgi-Cox stain. Note that apical and basal branches are relatively less in vehicle-treated 5xFAD mice and that SLCP treatment prevented this loss. **b**, **d** Representative dendritic spine images from apical and basal branches. **c**, **e** Morphometric data revealed that the number of dendritic spines were significantly decreased in vehicle-treated 5xFAD mice in comparison to their WT counterparts, whereas SLCP treatment mitigated these losses. **f** Representative images of layer II pyramidal neurons from entorhinal cortex. Fewer apical and basal branches were observed less in the vehicle-treated 5xFAD mice, but SLCP treatments mitigated this loss. **g**, **i** Representative dendritic spine images from apical and basal branches. **h**, **j** Dendritic spine density was significantly decreased in 5xFAD mice in comparison to WT mice, but SLCP treatment prevented much of this loss. **k** Representative images of apical and basal dendrites. l Representative images of primary, secondary, and tertiary dendrites from apical branch. Scale bar = 100 μm and is applicable to all images. *p < 0.05, **p < 0.01 in comparison to WT + vehicle, 5xFAD + SLCP, and WT + SLCP

**Fig. 5:**
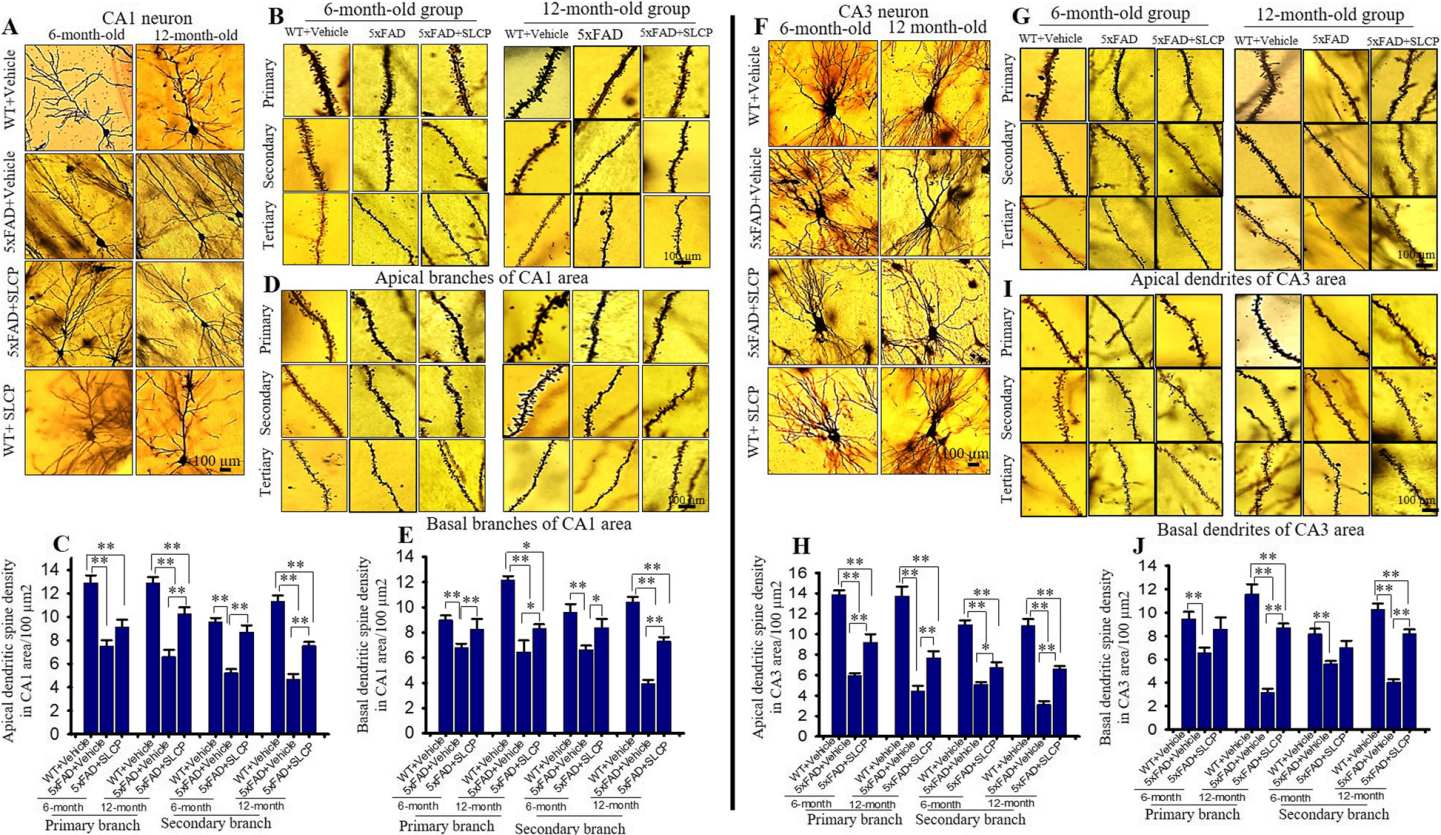
SLCP treatment partially preserved dendritic arborization and dendritic spine number in hippocampus of 5xFAD mice. After 2 months of treatment with SLCP (100 mg/kg), the brains of the 6- and 12-month-old 5xFAD and age-matched control mice were processed using Golgi-Cox stain for 2 weeks. Coronal sections (120 μm) were stained with 75% ammonium solution and 1% sodium thiosulphate. CA1 and CA3 neurons and dendritic spines from apical and basal branches (primary, secondary, and tertiary) were imaged using 40x and 100x objectives, respectively (Olympus). **a**, **f** Representative images of CA1 and CA3 pyramidal neurons showed a decreased number of apical and basal branches in vehicle-treated 5xFAD mice in comparison to WT and SLCP-treated mice. **b**, **d** Representative dendritic spine images from apical and basal branches. **c**, **e** Morphometric data revealed that the number of dendritic spines in CA1 neurons were significantly decreased in vehicle-treated 5xFAD mice in comparison to WT and SLCP-treated mice. **g**, **i** Representative dendritic spine images from CA3 apical and basal branches, respectively. **h**, **j** Morphometric analyses showed that the number of dendritic spines were significantly decreased in vehicle-treated 5xFAD mice in comparison to their WT counterparts, and to SLCP-treated 5xFAD mice. Scale bar = 100 μm and is applicable to all images. *p < 0.05, **p < 0.01 in comparison to WT + vehicle, 5xFAD + SLCP, and WT + SLCP

Below are the corrected Figs. 2, 4 and 5.
